# GPR40 full agonism exerts feeding suppression and weight loss through afferent vagal nerve

**DOI:** 10.1371/journal.pone.0222653

**Published:** 2019-09-16

**Authors:** Hikaru Ueno, Ryo Ito, Shin-ichi Abe, Hitomi Ogino, Minoru Maruyama, Hirohisa Miyashita, Yasufumi Miyamoto, Yusuke Moritoh, Yoshiyuki Tsujihata, Koji Takeuchi, Nobuhiro Nishigaki

**Affiliations:** 1 Cardiovascular Metabolic Drug Discovery Unit, Takeda Pharmaceutical Company Limited, Kanagawa, Japan; 2 Research and Development Division, SCOHIA PHARMA Inc., Kanagawa, Japan; CRCHUM-Montreal Diabetes Research Center, CANADA

## Abstract

GPR40/FFAR1 is a Gq protein-coupled receptor expressed in pancreatic β cells and enteroendocrine cells, and mediates insulin and incretin secretion to regulate feeding behavior. Several GPR40 full agonists have been reported to reduce food intake in rodents by regulating gut hormone secretion in addition to their potent glucose-lowering effects; however, detailed mechanisms of feeding suppression are still unknown. In the present study, we characterized T-3601386, a novel compound with potent full agonistic activity for GPR40, by using *in vitro* Ca^2+^ mobilization assay in Chinese hamster ovary (CHO) cells expressing *FFAR1* and *in vivo* hormone secretion assay. We also evaluated feeding suppression and weight loss after the administration of T-3601386 and investigated the involvement of the vagal nerve in these effects. T-3601386, but not a partial agonist fasiglifam, increased intracellular Ca^2+^ levels in CHO cells with low *FFAR1* expression, and single dosing of T-3601386 in diet-induced obese (DIO) rats elevated plasma incretin levels, suggesting full agonistic properties of T-3601386 against GPR40. Multiple doses of T-3601386, but not fasiglifam, in DIO rats showed dose-dependent weight loss accompanied by feeding suppression and durable glucagon-like peptide-1 elevation, all of which were completely abolished in *Ffar1*^*-/-*^ mice. Immunohistochemical analysis in the nuclei of the solitary tract demonstrated that T-3601386 increased the number of c-Fos positive cells, which also disappeared in *Ffar1*^*-/-*^ mice. Surgical vagotomy and drug-induced deafferentation counteracted the feeding suppression and weight loss induced by the administration of T-3601386. These results suggest that T-3601386 exerts incretin release and weight loss in a GPR40-dependent manner, and that afferent vagal nerves are important for the feeding suppression induced by GPR40 full agonism. Our novel findings raise the possibility that GPR40 full agonist can induce periphery-derived weight reduction, which may provide benefits such as less adverse effects in central nervous system compared to centrally-acting anti-obesity drugs.

## Introduction

Impaired insulin secretion from pancreatic β cells and peripheral insulin resistance are common characteristics observed in patients with type 2 diabetes **[1]**. Because obesity is one of the well-known risk factors of type 2 diabetes **[2–4]**, simultaneous control of both glycemic index and body weight (BW) is recommended.

GPR40/FFAR1 is a member of the 7-transmenbrane G protein-coupled receptors (GPCR), and medium- to long-chain fatty acids have been identified as its endogenous ligands **[5, 6]**. It is highly expressed in pancreatic β cells and agonist stimulation leads to elevations in intracellular Ca^2+^ levels followed by glucose-dependent insulin secretion **[7, 8]**. A synthetic GPR40 agonist, fasiglifam (TAK-875) **[9]** was firstly demonstrated to be effective in type 2 diabetic rodents and patients **[10–13]**. For several years, synthesized full agonists for GPR40 have been reported to evoke not only robust insulin secretion but also incretin release (glucagon-like peptide-1: GLP-1 and glucose-dependent insulinotropic polypeptide: GIP) from endocrine cells in the gastrointestinal (GI) tract **[14–17]**. As secreted GLP-1 elicits an anorectic effect **[18, 19]**, GPR40 full agonists might represent a novel therapeutic agent for the treatment of both diabetes and obesity. Although many previous studies related to GPR40 agonists have focused on their anti-diabetic actions, Gorski JN. *et al*. recently reported GPR40 agonist-induced suppression of food intake and BW **[20]**. Their results demonstrated that GLP-1 receptor (GLP-1R) as well as GPR40 contributed to the agonist-mediated feeding suppression; however, detailed analysis of its action site has not been carried out.

Feeding behavior is regulated by direct action of peptides and hormones on the central nervous system (CNS) and/or periphery-derived signals **[21]**. To link peripheral GI signals to feeding behavior, the afferent vagal nerves and the nucleus of the solitary tract (NTS) located in the brain stem play important roles **[22, 23]**. GI hormones such as GLP-1 and cholecystokinin (CCK) have been known to induce NTS activation and their anorectic effects were abolished in rodents with capsaicin-treated deafferentation or surgical vagotomy **[24–27]**, which suggests that food intake inhibition induced by these hormones requires intact vagal nerves and subsequent NTS activation. It is also possible that a GPR40 full stimulation evokes afferent vagal nerves and NTS activation by the release of endogenous GLP-1, and an eventual feeding suppression. However, the involvement of the vagal pathway in feeding behavior regulated by a GPR40 agonist has not been examined.

In this report, we first characterized 3-cyclopropyl-3-(3-((1-(2-(4,4-dimethylpentyl)-5-methoxyphenyl)piperidin-4-yl)methoxy)phenyl)propanoic acid (T-3601386), a racemic compound which clearly induces GPR40 full agonism **[28]**. Second, we examined the anti-obesity effect of T-3601386 in a diet-induced obese (DIO) model and conducted c-Fos immunohistochemistry in the NTS after T-3601386 administration, followed by the confirmation of their GPR40 dependence using *Ffar1*^*-/-*^ mice. Finally, we investigated the contribution of the vagal pathway to T-3601386-induced feeding suppression using *in vivo* models with vagal nerve blockade. In the present study, we demonstrated that T-3601386 shows potent *in vitro* and *in vivo* full agonistic properties against GPR40. Moreover, we concluded that afferent vagal nerves are activated in a GPR40-dependent manner and are involved in the feeding suppression elicited by a GPR40 full agonism.

## Materials and methods

### Materials

T-3601386, AM-1638 **[14, 15]** and fasiglifam were synthesized at Takeda Pharmaceutical Company, Limited. (Kanagawa, Japan). Alpha-linolenic, linoleic, palmitic and γ-linolenic acid were purchased from FUJIFILM Wako (Osaka, Japan), Cayman Chemical (Ann Arbor, MI) or Sigma (St Louis, MO). Sibutramine, liraglutide, and CCK-8 were purchased from Alexis Biochemicals (San Diego, CA), Novo Nordisk pharma (Tokyo, Japan), and Peptide Institute, Inc. (Osaka, Japan), respectively. Compounds were dissolved in dimethyl sulfoxide for the *in vitro* study and were suspended or dissolved in 0.5% methylcellulose solution (FUJIFILM Wako) for oral dosing. Liraglutide and CCK-8 were dissolved in saline.

### Animals

Male F344 and SD rats were purchased from CLEA Japan, Inc. (Tokyo, Japan) and Charles river Laboratories Japan, Inc. (Yokohama, Japan), respectively. Male neonatally streptozotocin-induced diabetic rats (N-STZ-1.5 rats) **[29]**, male *Ffar1*^*-/-*^, age-matched littermate (wild-type) mice **[30]** and female Wistar fatty rats **[31]** were obtained from RABICS, LTD. (Kanagawa, Japan). All rats and mice were housed in individual cages in a room with controlled temperature (23°C), humidity (55%) and lighting (lights on from 7:00 am to 7:00 pm) and were allowed free access to solid or powdered standard laboratory chow diet (CE-2, CLEA) and tap water. The animals were fed a high fat diet (HFD; D12451, D12451M, Research diets, Inc., New Brunswick, NJ) if needed as described below. The care and use of the animals and experimental protocols used in this study were approved by the IACUC of Shonan Research Center, Takeda Pharmaceutical Company Limited (Japan). Animals were monitored every day to check their general appearance. Their health and well-being were assessed by the following metrics: general activity, body temperature, trauma, tumor, tremors, breathing, dehydration and BW. To minimize animal suffering and distress, an appropriate treatment of analgesics and anesthetics was performed as described in each of the sections below. Euthanasia was conducted via carbon dioxide gas.

### *In vitro* Ca^2+^ mobilization assay

Chinese hamster ovary (CHO) cells stably expressing human *FFAR1* with different receptor mRNA expression levels (clones #104 and #2) **[32]** were cultured with minimum essential medium-alpha (FUJIFILM Wako) containing 10% dialyzed fetal bovine serum (FBS, GE Healthcare Life Sciences, Buckinghamshire, UK), 10 mM HEPES (Thermo Fisher Scientific, Waltham, MA), 100 IU/mL penicillin, and 100 μg/mL streptomycin in 5% CO_2_ at 37°C. Cells (1 x 10^4^ cells /well) were seeded in 384 well plates and then incubated overnight. After removing of the medium, cells were incubated in 30 μL of loading buffer (Hanks' Balanced Salt Solution containing 20 mM HEPES, 0.1% fatty acid-free BSA (FUJIFILM Wako), 0.08% Pluronic F127 (#CSK-01F, Dojindo Kumamoto, Japan), 2.5 mmol/L Probenecid (#CSK-03F, Dojindo) and 2.5 μg/mL Fluo4 (#F311, Dojindo)) for 60 min in 5% CO_2_ at 37°C. Test compounds at various concentrations were added into the cells and increase of the intracellular Ca^2+^ concentration was monitored by the FLIPR Tetra system (Molecular Devices, Tokyo, Japan) for 180 sec. EC_50_ was calculated by data analysis using a 4-parameter logistic equation in Graphpad Prism 7 software.

### *In vitro* GLP-1 secretion assay

GLUTag cells were cultured in Dulbecco's modified Eagle's medium (DMEM, high glucose; Invitrogen) with 10% heat-inactivated FBS (Thermo Fisher Scientific), 100 IU/mL penicillin and 100 μg/mL streptomycin in a humidified atmosphere containing 5% CO_2_ at 37°C. Cells (1 x 10^4^ cells/well) were seeded in 96 well poly-L-lysine coated plates (Sumitomo Bakelite, Tokyo, Japan). The following day, medium was replaced with low glucose DMEM prior to overnight incubation. After washing, Krebs-Ringer-bicarbonate HEPES buffer containing 0.2% fatty acid-free BSA and 10 mmol/L glucose with or without stimulators was added, followed by incubation for 2 h at 37°C. Secreted active GLP-1 concentration was measured using a GLP-1 Active form Assay Kit (27784, IBL, Gunma, Japan).

### *In vivo* hormone secretion by single dosing

F344 rats were fed HFD from 5 weeks old in order to develop diet-induced obesity (defined as DIO-F344 rats). DIO-F344 rats (41 weeks old, baseline BW 532 ± 16 g) were randomized based on their BW, and were bled from the tail vein at indicated time points after drug administration (N = 6 per group, total N = 18). Plasma total GLP-1 levels were determined using sandwich ELISA, which had been established by Takeda Pharmaceutical Company Limited **[33]**. Plasma total GIP levels were measured using a commercially available total GIP ELISA kit (EZRMGIP-55K, Merck Millipore, Burlington, MA, USA).

### Oral glucose tolerance test

Diabetic N-STZ-1.5 rats with impaired insulin secretion were used for oral glucose tolerance test **[33, 34]**. Overnight fasted rats (23 weeks old) were randomized based on plasma glucose, triglyceride and BW, and were administered drugs 1 h before oral glucose loading (1.5 g/kg) (N = 6 per group, total N = 18). Blood samples were obtained from the tail vein at indicated time points and plasma parameters were determined. Plasma glucose and triglyceride were measured using an Autoanalyzer 7180 (Hitachi, Tokyo, Japan), and plasma insulin was measured using a radioimmunoassay kit (RI-13K, Merck Millipore).

### Repeated dosing studies in DIO-F344 rats and *Ffar1*^*-/-*^ mice

DIO-F344 rats (42 weeks old, HFD for 37 weeks, baseline BW 480 ± 18 g) were randomized based on body fat mass measured by an EchoMRI-900 (Echo Medical Systems), BW and food intake, and were administered drugs once daily for 4 weeks (N = 6–7 per group, total N = 41). After the repeated doses, blood samples were collected from the tail vein at the indicated time points after a single further dosing and plasma total GLP-1 levels were measured as described above. *Ffar1*^*-/-*^ and wild-type mice (23 weeks old, baseline BW 45 ± 5 g for wild and 44 ± 5 g for *Ffar1-/-*) fed HFD for 16 weeks were randomized based on their BW and food intake, and were administered drugs once daily for 3 days (N = 5–6 per group, total N = 17 in each genotype). Blood samples were collected from the tail vein before and 1 h after the 4^th^ dosing and plasma total GLP-1 and GIP levels were measured as described above.

### c-Fos immunohistochemistry

SD rats (8 weeks old, baseline BW 291 ± 14 g) fed HFD for 2 weeks were randomized based on BW, and were administered drugs (N = 4 per group, total N = 16). Rats were dissected 3 h after the dosing to isolate the brain stem under sodium pentobarbital (50 mg/kg, *i*.*p*., Kyoritsu, Tokyo, Japan) anesthesia. The obtained brain samples were fixed with 4% paraformaldehyde for 24 h, followed by substitution with 30% sucrose in phosphate-buffered saline (PBS) for 2 days. Immunostaining was performed by a free-floating method. Forty μm cryosections including NTS were incubated with anti-cFos rabbit polyclonal antibody (SC-52; Santa Cruz Biotechnology) diluted 1:4000 in PBS at pH 7.1 containing 1% normal horse serum and 0.4% Triton X-100 overnight. Immunoreactivity in the sections was visualized using diaminobenzidine after the treatment with an ABC Elite kit (PK-6101, Vector Laboratories, Inc., Burlingame, CA). After processing, the sections were mounted on a non-coated slide, dehydrated, and then cover-slipped. Photographs were captured with a 4 x objective lens using Nikon ECLIPSE 800 microscope equipped with Nikon ACT-1 version 2.12. c-Fos positive cells were counted using a computerized image analysis system (Image-Pro Plus version 4.5). c-Fos counts were performed on each of three sections from each animal. The mean of these three determinations was used for subsequent statistical analysis. In the same protocol as described above, *Ffar1*^*-/-*^ and wild-type mice (46 weeks old, baseline BW 50 ± 5 g for wild and 55 ± 2 g for *Ffar1-/-*) fed HFD for 38 weeks were also used to confirm the GPR40 dependency (N = 5–6 per group, total N = 11 in each genotype).

### Surgical vagotomy

Overnight fasted SD rats (10 weeks old) pretreated with buprenorphine (0.01 mg/kg, *s*.*c*., Otsuka, Tokyo, Japan) were operated for bilateral subdiaphragmatic vagotomy under sodium pentobarbital (50 mg/kg, *i*.*p*.) anesthesia as previously reported **[35]**. Eleven days after the operation, the rats began a HFD feeding regime until the end of the study (3 weeks of HFD feeding). Four weeks after the operation, the rats were randomized based on their food consumption (Pre) and BW (561 ± 21 g for sham and 484 ± 62 g for vagotomy, N = 6 per group, total N = 12 in each surgery). The drugs were orally administered, and food consumption for 24 h was calculated (% change from Pre). The success of surgical vagotomy was confirmed by means of the significant cancellation of the CCK-8 (3 μg/kg, *i*.*p*.)-induced anorectic effect for 30 min in the overnight fasted-refed experiment **[26, 36]**.

### Drug-induced deafferentation

Wistar fatty rats (27 weeks old) pretreated with buprenorphine (0.01 mg/kg, *s*.*c*.) were administered a transient receptor potential vanilloid type 1 agonist, resiniferatoxin (RTX; 40 μg/kg, LC Laboratories, Woburn, MA) or vehicle (1% ethanol, 1% Tween80, 98% saline) subcutaneously under isoflurane anesthesia (DS pharma, Osaka, Japan). A week after the injection of RTX or vehicle, rats were randomized based on their food consumption (Pre) and BW (529 ± 27 g for vehicle and 516 ± 29 g for RTX, N = 6 per group, total N = 24 in each treatment). The drugs were orally administered and food consumption for 24 h was calculated (% change from Pre). The following week, drugs were administered, and blood samples were collected from the tail vein before and 1 h after dosing for measurements of plasma incretin. The success of deafferentation by RTX was confirmed by the disappearance of capsaicin (0.1 mg/mL, FUJIFILM Wako)-induced eye-wipe reaction **[37]**.

### Statistics

All data are expressed as the mean ± S.D. (*in vivo* experiments) or means ± S.E.M. (*in vitro* experiments). Statistical significance was determined by Student’s t-test or Aspin-Welch test between two groups depending on equal variances. Statistical significance for multiple comparisons was determined by Steel's test. The dose-dependent effects of drugs were analyzed by one-tailed Williams' test or Shirley-Williams test. Two-way ANOVA was performed to evaluate combinational effects. When an interaction effect was significant (p<0.05), the combinational effect was interpreted to be meaningful (the effects of combination treatment exceeds the sum of each effect) **[11]**.

## Results

### T-3601386 shows full agonistic activities for GPR40

**Fig 1:**
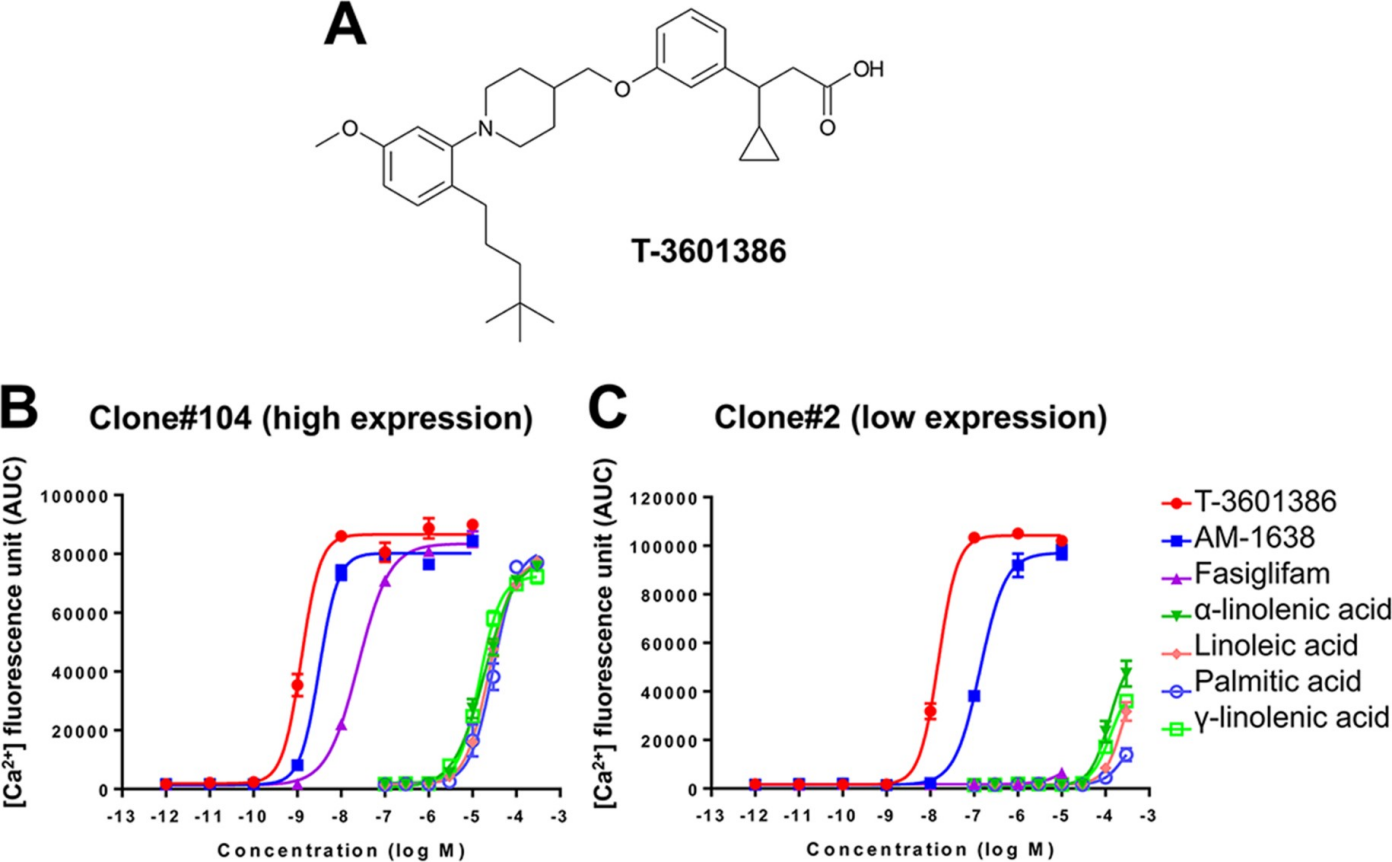
Chemical structure of T-3601386 and *in vitro* activities in CHO cells. **(A)** The chemical structure of T-3601386. **(B, C)** Intracellular Ca^2+^ concentrations were measured before and after adding samples using FLIPR in CHO cells with high (clone#104) **(B)** and low expression (clone#2) **(C)** of human *FFAR1*. Each data point represents the mean ± S.E. (quadruplicate). Comparable results were obtained by another independent experiment.

**Fig 2:**
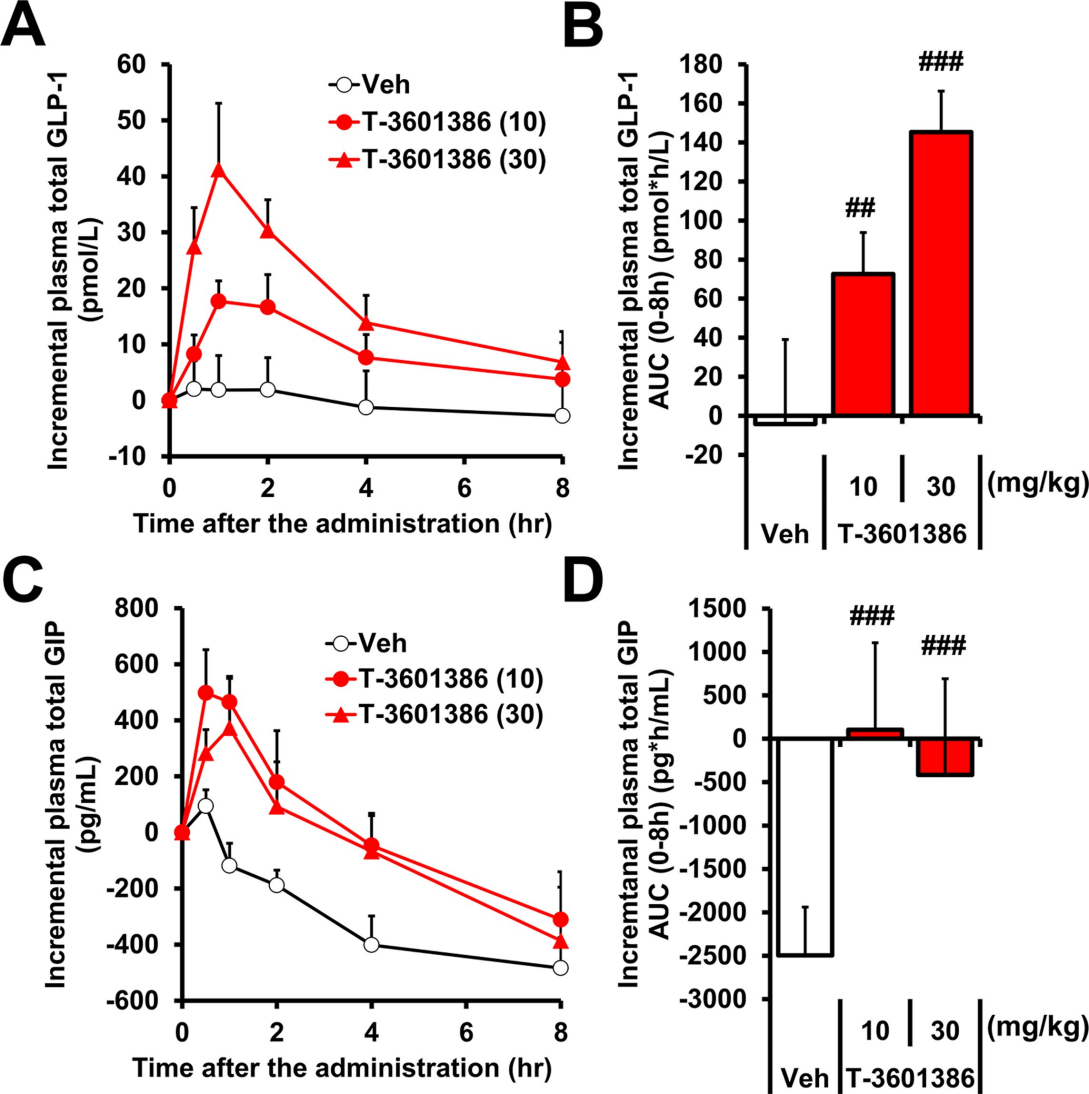
Effects of T-3601386 on *in vivo* incretin secretion in DIO-F344 rats. Vehicle or T-3601386 (10 or 30 mg/kg) were orally administered, and plasma level of total GLP-1 and GIP were monitored in DIO-F344 rats. Graphs showed the time-dependent changes of incremental plasma levels of total GLP-1 **(A)** and GIP **(C)** after drug administration. Areas under the curve of incremental plasma levels of total GLP-1 and GIP (0–8 h) were shown in **(B)** and **(D)**, respectively. Veh; vehicle. Each data point represents the mean ± S.D. (N = 6). ##p<0.005, ###p<0.0005 vs. vehicle by one-tailed Williams' test.

It has been previously reported that in Ca^2+^ mobilization assays in CHO cells with relatively high (clone #104) and low (clone #2) expression of human *FFAR1*, maximal response to the partial agonist fasiglifam dramatically decreases compared with an endogenous full agonist as expression levels of GPR40 decrease, indicating that partial and full agonists can be distinguished using this assay system **[32]**. We evaluated the GPR40 agonistic activities of T-3601386 **(Fig 1A)**, AM-1638 (a well-known full agonist), fasiglifam (a partial agonist) and several free fatty acids (endogenous full agonists) on Ca^2+^ mobilization in the same 2 clones of CHO cells. While both T-3601386 and AM-1638 showed concentration-dependent increases in the intracellular Ca^2+^ mobilization in both clones (#104 and #2), fasiglifam increased intracellular Ca^2+^ only in high expressing clone #104. Free fatty acids also showed agonist activities in both clones only at high concentrations **(Fig 1B and 1C)**. EC_50_ values (clone #104/#2) of tested compounds were as follows: 1.2/15, 3.2/138, 25/>1000 and >10000/>10000 nmol/L (T-3601386, AM-1638, fasiglifam, and free fatty acids, respectively). In clone #2 CHO cells, the E_max_ value of T-3601386 (246%, % γ-linolenic acid) was comparable to and higher than that of AM-1638 (229%) and fasiglifam (15%), respectively. To further characterize the full agonistic property of T-3601386, the effects of T-3601386 on GLP-1 secretion were examined in mouse enteroendocrine cells. T-3601386 elevated GLP-1 secretion in GLUTag cells at 10-fold lower concentration compared with AM-1638 **(S1 Fig)**. Next, we tested *in vivo* incretin secretion and clear increases in plasma levels of total GLP-1 and GIP were observed after oral dose of T-3601386 (10 and 30 mg/kg) in DIO-F344 rats **(Fig 2A–2D)**. Moreover, the robust improvement of glucose tolerance and enhanced insulin secretion after a single dose of T-3601386 were also confirmed in diabetic N-STZ-1.5 rats **(S2 Fig)**. These results indicate that T-3601386 also elicits *in vivo* GPR40 full agonism. After the confirmation of these strong full agonistic *in vitro* and *in vivo* activities for GPR40, we performed subsequent experiments using T-3601386.

### T-3601386 exerts anorectic and BW-lowering effects with multiple dosing

**Fig 3:**
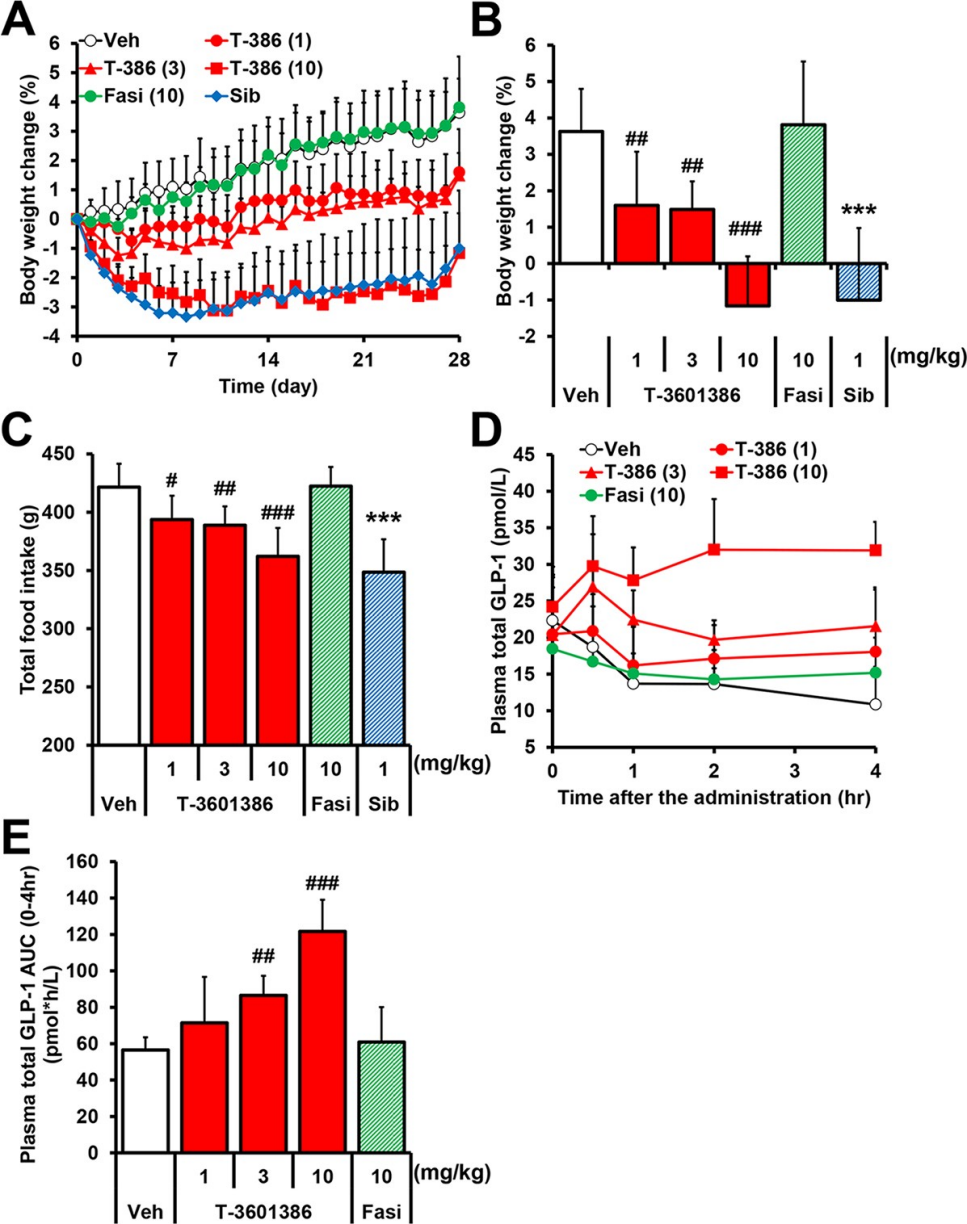
Effects of repeated administration of T-3601386 in DIO-F344 rats. Four weeks multiple dosing of vehicle or T-3601386 (1, 3, 10 mg/kg), fasiglifam (10 mg/kg) and sibutramine (1 mg/kg) was performed in DIO-F344 rats. BW **(A)** and food intake were monitored continuously over a period of 4 weeks. The BW change at the end of experiment **(B)** and cumulative food consumption **(C)** were calculated. **(D, E)** Graphs showed time-dependent changes **(D)** and the area under the curve (0–4 h) **(E)** of the plasma levels of total GLP-1 by the further single dose after the repeated doses. Veh; vehicle, T-386; T-3601386, Fasi; fasiglifam, Sib; sibutramine. Each data point represents the mean ± S.D. (N = 6 for vehicle and N = 7 for the other groups). #p<0.025, ##p<0.005, ###p<0.0005 vs. vehicle by one-tailed Williams' test or Shirley-Williams test, ***p<0.001 vs. vehicle by Student’s t-test.

The BW of rats fed HFD (D12451) kept increasing after HFD feeding, and took over 16 weeks to reach a plateau **[38]**. In addition, several anti-obese compounds have been tested using DIO rats fed HFD for long term (27–46 weeks) **[39, 40]**. To precisely evaluate weight-reducing effects of T-3601386, we selected DIO rats fed HFD for long term especially in multiple dosing study. Four weeks multiple treatment with T-3601386 (1, 3 and 10 mg/kg), but not fasiglifam (10 mg/kg), dose-dependently and significantly reduced food consumption and BW in DIO-F344 rats **(Fig 3A–3C)**, and the potency of T-3601386 at 10 mg/kg (-4.8% vs. vehicle) was comparable to that of sibutramine (1 mg/kg) (-4.6% vs. vehicle), a centrally-acting anti-obesity drug, as a positive control. In DIO-F344 rats given T-3601386 for 4 weeks, significant increases in plasma GLP-1 levels were observed following the single dose of T-3601386 **(Fig 3D and 3E)**, suggesting the continuous elevation of plasma GLP-1 levels during the repeated dosing study. These results are consistent with a previous report that a GPR40 full agonist exerts BW-lowering effect with the augmentation of circulating GLP-1 **[16]**.

### T-3601386 administration induces NTS activation

**Fig 4:**
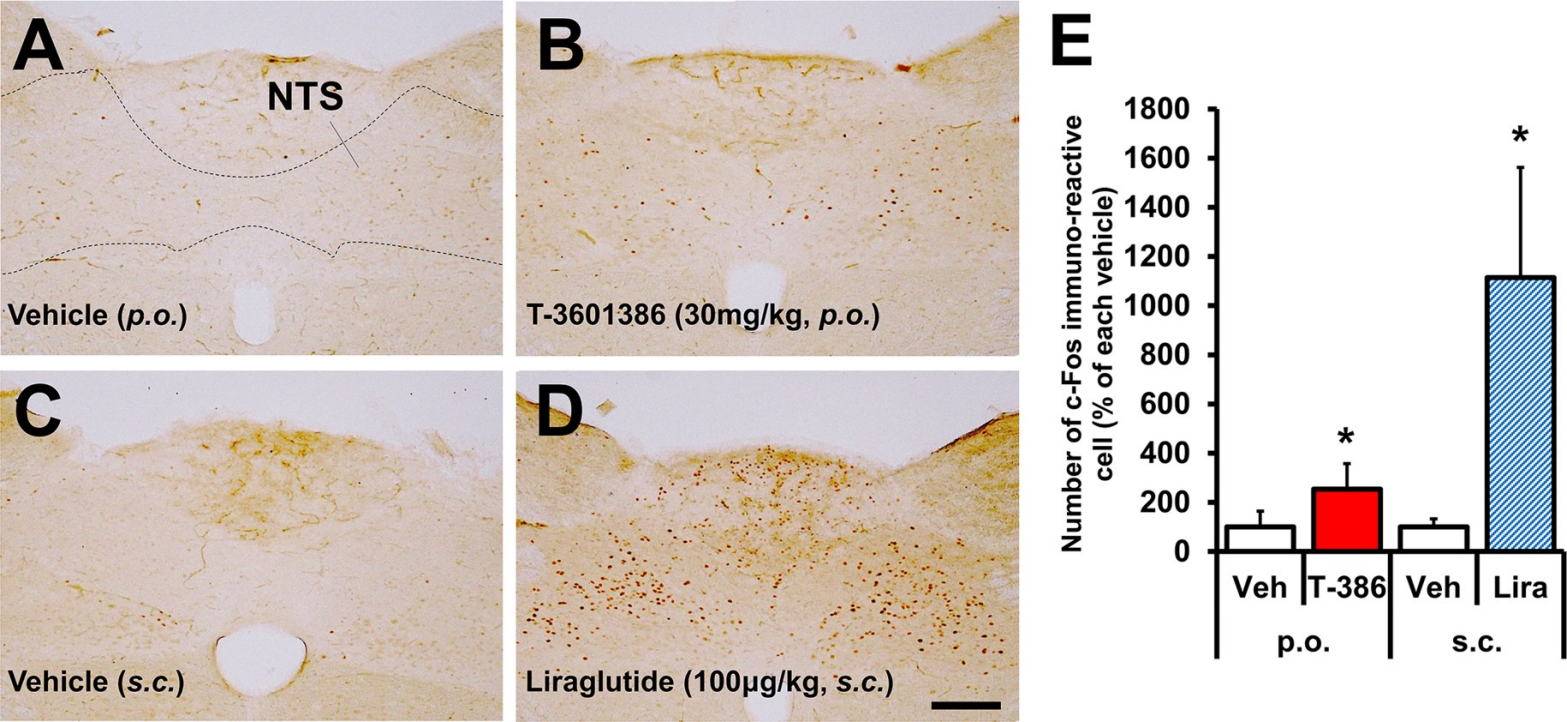
Effects of T-3601386 on c-Fos immunoreactivity in NTS. SD Rats were dissected to obtain the brain stem regions 3 h after the oral or subcutaneous drug administration. The immunohistochemical images for c-Fos staining after the administration of vehicle (*p*.*o*.) **(A)**, T-3601386 (*p*.*o*.) **(B)**, vehicle (*s*.*c*.) **(C)** and liraglutide (*s*.*c*.) **(D)** were shown. The location of NTS in the brain section was shown in **(A)** according to the brain atlas **[41]**. c-Fos immunoreactive cells in NTS were automatically counted for their quantification as percentage of each vehicle **(E)**. Bar: 200 μm. Veh; vehicle, T-386; 30 mg/kg of T-3601386, Lira; 100 μg/kg of liraglutide. Each data point represents the mean ± S.D. (N = 4). *p<0.05 vs. each vehicle by Student’s t-test or Aspin-Welch test.

As mentioned above, many reports have demonstrated that GLP-1 and other GI hormones require the intact vagal nerve for their food intake suppression **[24–27]**. We hypothesized that elevated GLP-1 by a GPR40 full agonism can activate NTS, which receives peripheral projection via the afferent vagal nerve **[22–23]**. In this experiment, we adopted rats that had been fed a HFD for a relatively short amount of time instead of using completely established DIO rats with long term HFD feeding for reasons described in the vagal nerve blockade result section. A single dose of T-3601386 (30 mg/kg) in rats showed a mild but statistically significant increase in c-Fos immunoreactivity in the NTS **(Fig 4A, 4B and 4E) [41]**, and the GLP-1 analogue liraglutide (100 μg/kg, *s*.*c*.) as a positive control also resulted in a marked increase in c-Fos immunoreactive cells in the NTS **(Fig 4C–4E)**, suggesting that the afferent vagal pathway became activated after the administration of T-3601386.

### Feeding suppression, incretinotropic effects, and NTS activation induced by T-3601386 are dependent on GPR40

**Fig 5:**
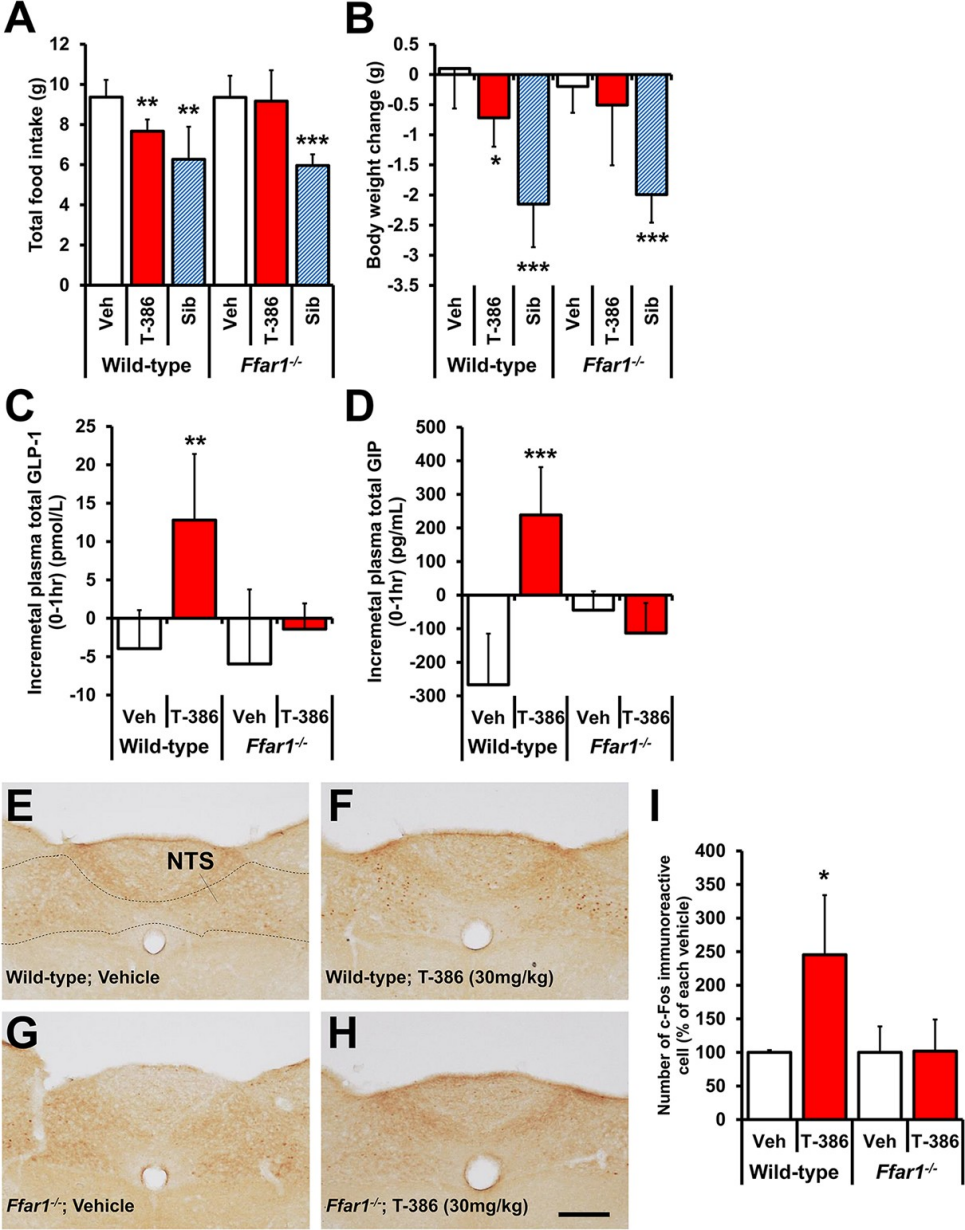
Effects of T-3601386 on food intake, BW, plasma incretin levels and NTS activation in *Ffar1*^*-/-*^ and wild-type mice fed HFD. **(A-D)** Vehicle, T-3601386 (30 mg/kg) or sibutramine (10 mg/kg) was orally administered for 3 days in *Ffar1*^*-/-*^ and wild-type mice, and the cumulative food consumption **(A)** and BW change at the end of experiment **(B)** were calculated. The plasma levels of total GLP-1 **(C)** and GIP **(D)** before and 1 h after the 4^th^ drug administration were measured, and the incremental values of those hormones levels were calculated. Each data point represents the mean ± S.D. (N = 5 for sibutramine and N = 6 for the other groups). **(E-I)** *Ffar1*^*-/-*^ and wild-type mice were dissected to obtain the brain stem regions 3 h after the oral drug administration. The immunohistochemical images for c-Fos staining after the administration of vehicle **(E)**, T-3601386 **(F)** in wild-type mice, vehicle **(G)** and T-3601386 **(H)** in *Ffar1*^*-/-*^ mice were shown. The location of NTS in the brain section was shown in **(E)**. c-Fos immunoreactive cells in NTS were automatically counted for their quantification as percentage of each vehicle **(I)**. Each data point represents the mean ± S.D. (N = 5 for each vehicle and N = 6 for the other groups). Bar: 200 μm. Veh; vehicle, T-386; 30 mg/kg of T-3601386, Sib; 10 mg/kg of sibutramine. *p<0.05, **p<0.01, ***p<0.001 vs. each vehicle by Student’s t-test or Aspin-Welch test.

To confirm the involvement of GPR40 in T-3601386-induced feeding suppression and BW loss, the effects of T-3601386 in *Ffar1*^*-/-*^ and wild-type mice were evaluated. Three daily doses of T-3601386 (30 mg/kg) significantly reduced food intake in wild-type mice, but not in *Ffar1*^*-/-*^ mice (p = 0.81) **(Fig 5A)**. Similarly, a significant decrease in BW was observed only in wild-type mice, although T-3601386 showed slight, but not significant reduction in BW in *Ffar-/-* mice (p = 0.51) **(Fig 5B)**. Comparable decreases in food intake and BW were observed both in wild-type and *Ffar1*^*-/-*^ mice given sibutramine (10 mg/kg). After 3 days of administration, the significant elevation of plasma incretin after the dosing of T-3601386 in wild-type mice disappeared in *Ffar1*^*-/-*^ mice (p = 0.32 and p = 0.15 in GLP-1 and GIP, respectively) **(Fig 5C and 5D)**. A GPR40 dependence of T-3601386-induced NTS activation was also examined by immunohistochemistry. As observed in rats, a single dosing of T-3601386 (30 mg/kg) significantly increased c-Fos immunoreactive cells in the NTS of wild-type mice **(Fig 5E, 5F and 5I)**, but this effect completely disappeared in *Ffar1*^*-/-*^ mice (p = 0.94) **(Fig 5G–5I)**.

### Vagal nerve blockade attenuates T-3601386-induced feeding suppression and weight loss

**Fig 6:**
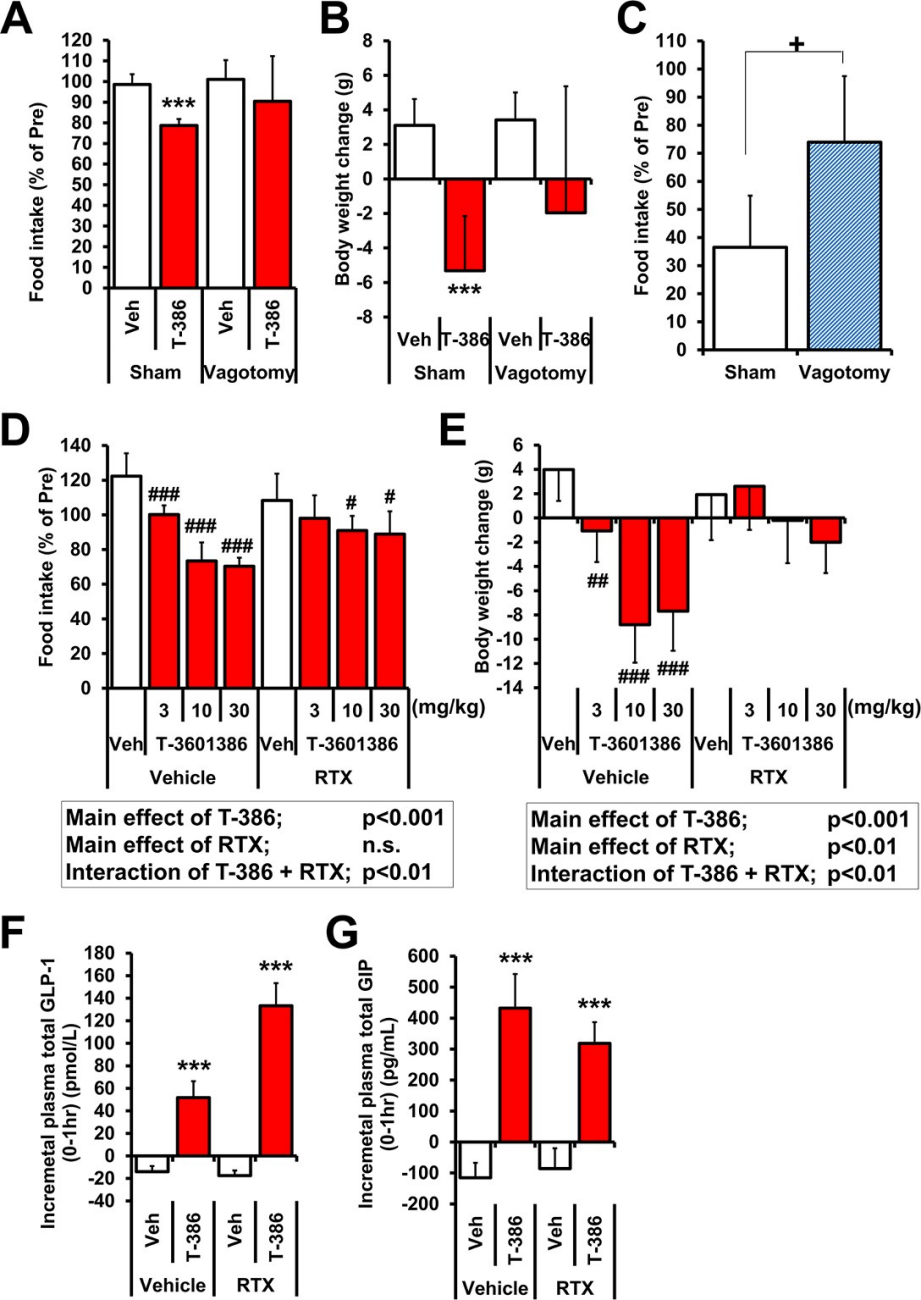
Effects of T-3601386 on food intake and BW in rats with vagal nerve blockade. Vehicle or T-3601386 (30 mg/kg) were orally administered in vagotomized or sham-operated rats, and the food consumption for 24 h **(A)** and BW change **(B)** were calculated. As a positive control, CCK-8 (3 μg/kg, *i*.*p*.)-induced anorectic effects for 30 min in overnight fasted vagotomized or sham-operated rats were shown **(C)**. Vehicle or T-3601386 (3, 10, 30 mg/kg) was administered orally to female Wistar fatty rats w/wo RTX treatment, and the food consumption for 24 h **(D)** and BW change **(E)** were calculated. The plasma levels of total GLP-1 **(F)** and GIP **(G)** before and 1 hour after the single administration of vehicle or T-3601386 (10 mg/kg) were measured, and the incremental values of those hormone levels were calculated. The results of two-way ANOVA in **(D)** and **(E)** were shown in each inset. Veh; vehicle, T-386; 30 **(A, B)** or 10 mg/kg **(F, G)** of T-3601386. Each data point represents the mean ± S.D. (N = 6). #p<0.025, ##p<0.005, ###p<0.0005 vs. vehicle by one-tailed Williams' test, ***p<0.001 vs. each vehicle by Student’s t-test or Aspin-Welch test, +p<0.05 vs. Sham by Student’s t-test.

To clarify the involvement of the vagal nerve in the feeding suppression of T-3601386, we evaluated the effects of T-3601386 in two models with surgical and pharmacological vagal nerve blockade, namely bilateral subdiaphragmatic vagotomy and RTX-induced deafferentation, respectively. As was the case in the c-Fos immunostaining experiment **(Fig 4)**, surgical vagotomy was performed using relatively short term HFD regimen because it was difficult to perform surgery in animals with severe obesity and abundant abdominal fat (such as established DIO rats). Moreover, we thought that a similarly designed protocol had to be applied to the experiments of c-Fos immunostaining **(Fig 4)** and vagotomy to associate vagal nerve activation with vagally-mediated feeding suppression. Based on these reasons, both of these experiments needed to be conducted using similar short term HFD regimens. Significant decreases in food intake (20% inhibition vs. control) and BW with T-3601386 (30 mg/kg) were observed in sham-operated rats, while T-3601386 administration in vagotomized rats showed relatively weak efficacy both in feeding suppression (11% inhibition vs. control, p = 0.31) and weight loss (p = 0.13) **(Fig 6A and 6B)**. To demonstrate the success of the operation, we used CCK-8 which has been known to reduce food intake through intact vagal nerves as mentioned above. CCK-8-induced anorectic effect in sham-operated rats was significantly attenuated in vagotomized rats, indicating the successful surgical vagotomy in the present study **(Fig 6C)**. To demonstrate further universality and reproducibility of GPR40 full agonism-derived feeding suppression, we finally investigated the effects of T-3601386 in genetically-obese rats and a counteraction of pharmacological (RTX-treated) deafferentation on T-3601386-induced *in vivo* effects. Wistar fatty rats show a highly-obese phenotype without HFD feeding due to a mutation in the leptin receptor gene **[31]**. T-3601386 dose-dependently and significantly decreased food consumption in vehicle-treated Wistar fatty rats (% inhibition vs. control: 18, 40 and 43% in 3, 10 and 30 mg/kg, respectively) **(Fig 6D)**. However, in rats treated with RTX, which induces afferent-specific denervation **[37]**, the effects of T-3601386 were markedly attenuated (% inhibition vs. control: 9, 16 and 18% in 3, 10 and 30 mg/kg, respectively) **(Fig 6D)**. The significant BW-lowering effect of T-3601386 in vehicle-treated rats also disappeared in RTX-treated rats **(Fig 6E)**. The results of a two-way ANOVA showed the significant interaction effects between T-3601386 and RTX (p<0.01 both in food intake and BW) **(Fig 6D and 6E)**, which precisely and strongly supported the impaired response of T-3601386 after the treatment of RTX. On the other hand, elevations of plasma GLP-1 and GIP after the dosing of T-3601386 were not attenuated in RTX-treated rats **(Fig 6F and 6G)**. Successful deafferentation by RTX treatment was confirmed by the disappearance of the capsaicin-induced eye-wipe reaction through the whole of this experiment.

## Discussion

Fasiglifam, a partial agonist of GPR40, has been shown to be effective in patients with type 2 diabetes **[12, 13]**. As a novel orally-available drug, a GPR40 full agonist is expected to exert not only anti-diabetic but also BW-lowering effects through its incretin secretory activity in addition to a potent insulinotropic effect. In the present study, we showed that T-3601386 clearly reduces food intake and BW accompanied by incretin secretion via GPR40. In addition, we also newly found that GPR40 full agonism induces an activation of afferent vagal nerves, which play an important role in full agonism-elicited feeding suppression and weight loss.

T-3601386 showed stronger *in vitro* activity than a partial agonist, fasiglifam, and a full agonist, AM-1638, in CHO cells and GLUTag cells. Next, *in vivo* studies demonstrated that T-3601386 has GPR40-dependent incretinotropic capacity and a beneficial effect on obesity. These results indicated that T-3601386 is a potent and orally available compound showing full agonistic activity for GPR40. Moreover, we have shown that T-3601386 reduces food intake and BW not only in HFD-feeding obese rats and mice but also in genetically-obese rats, suggesting a clear universality of GPR40 full stimulation-derived feeding suppression. Recent report by Gorski JN. *et al*. indicated that GPR40 full agonist-induced food intake suppression requires GLP-1R activation **[20]**, and in the present study, a single dose of T-3601386 elevated plasma levels of total GLP-1, even after multiple treatments, with no desensitization. From these observations, we speculate that secreted GLP-1 lends major contributions to the durable BW reduction induced by T-3601386. Although we also confirmed *in vivo* GIP elevation to be one of the hallmarks of GPR40 full agonism, GIP levels were decreased over time in vehicle-treated rats **(Fig 2C)**. GIP is known to be secreted in response to fat-rich meals **[42]**, and plasma levels of GIP were reported to be affected by meals to a larger extent than those of GLP-1 **[43]**. In our present study, since plasma samples were collected during the light period, a reduced feeding phase for rats, basal GIP levels might have gradually decreased.

In the GI tract, GPR40 has been reported to co-localize not only with GLP-1 and GIP but also with other intestinal hormones **[44]**, and in fact, peptide YY (PYY) secretion was observed after dosing with other full agonists **[16, 17]**. Previous reports suggested that a novel strategy through dual receptor agonism of GLP-1/GIP or GLP-1/PYY would lead to a greater BW loss with anti-diabetic effects than each mono-hormonal therapy **[45, 46]**. These results raise the possibility that GIP and/or PYY secreted by T-3601386 potentiate food intake suppression by GLP-1, and that eventually T-3601386 may behave as an indirect agonist for endogenous dual/triple (or more) receptors. Throughout the study, changes in BW after the administration of T-3601386 were relatively well correlated with changes in food intake, suggesting that weight reduction would be mainly caused by food reduction, but not via enhancement of energy expenditure. On the other hand, a GPR40 full agonist also increases plasma levels of glucagon **[16]**, which is well known to increase energy expenditure. Our recent publication also reported on glucagonotropic action that occurs as a result of GPR40 full stimulation **[33]**. The potential role of glucagon in the weight-reducing effects of T-3601386 should be assessed in a future study.

The elevation of c-Fos immunoreactivity after the dosing of T-3601386 in the present study is the first evidence for NTS activation following GPR40 agonism. NTS activation is known to result from the stimulation of afferent vagal nerve **[22, 23]**, indicating that T-3601386 interacts with the afferent vagal nerve though unsolved, but GPR40-dependent, mechanisms. Regarding the direct action on vagal nerve, however, almost no expression of GPR40 was reported in vagal afferents compared with the well-known receptors for GLP-1, PYY and CCK **[47]**, indicating the low probability of direct stimulation of GPR40 on the vagal nerve. With respect to indirect effects via metabolic hormones, we have recently demonstrated that GLP-1, GIP, PYY, glucagon and insulin are secreted after *in vivo* GPR40 full agonist stimulation **[33]**. Among them, similarly with GPR40, the expressions of GIP and glucagon receptors are not observed in vagal afferents **[47]**. Regarding insulin, although some studies reported the expression of insulin receptor in nodose ganglia **[48, 49]**, it remains unclear whether insulin-stimulated vagal afferents can transmit the signal to the NTS/brain and influence eventual physiological effects as GLP-1R lignads can. In our present study, we demonstrated clear *in vivo* NTS activation after dosing of T-3601386 in a GPR40-dependent manner, representing stark evidence for signal transmission from peripheral drug administration leading to activity changes in the brain stem. All of this being considered, it is reasonable to surmise that NTS neurons were activated mainly by secreted GLP-1 (and PYY unmeasured in this study) after GPR40 full stimulation. Of course, we cannot conclude that the elevation of GLP-1, rather than insulin, contributed to NTS activation until we conduct experiments using GLP-1R KO mice and/or GLP-1R antagonists as future studies.

The results in rats with surgical vagotomy and RTX-induced deafferentation clearly showed the involvement of the afferent vagal nerve in feeding suppression and weight loss with T-3601386. Considering at least no decreases in plasma GLP-1 and GIP levels with T-3601386, the peripheral incretin secretion normally occurred via GPR40 even after deafferentation, and subsequent transmission of the anorectic signal to the brain through the vagal nerve was seemingly disrupted by vagal nerve blockade. As mentioned above, we speculate that the possible main factor for vagally-mediated feeding suppression by T-3601386 would be GLP-1. This hypothesis can be supported by our denervation experiments and the previous findings of high GLP-1R expression on vagal afferents **[47]**, GLP-1R-related appetite suppression through the vagal nerve **[24, 25, 50]** and the disappearance of GPR40-derived food reduction by GLP-1R depletion **[20]**. The incomplete suppression of T-3601386-induced feeding suppression in models with vagal nerve blockade suggests the existence of other circulating factors delivered to the brain via non-neuronal pathways. Although more detailed mechanisms remain to be elucidated, the afferent vagal nerves, at least in part, contribute to GPR40 full agonism-induced feeding suppression and BW loss, which are probably mediated by secreted metabolic hormones.

In conclusion, our synthetic compound, T-3601386, exerts BW loss associated with its feeding suppression via GPR40. Moreover, we found for the first time that GPR40 full agonism evokes the activation of the vagal nerve-NTS pathway, which plays an important role for feeding suppression and weight loss. Our present study provides a novel aspect of GPR40 pharmacology in feeding regulation and raises the possibility that a GPR40 full agonist may become a weight-reducing drug with less CNS adverse effects, since vagally-mediated weight reduction does not always require drug exposure to the CNS.

## Supporting information

S1 Fig *in vitro* GLP-1 secretion in GLUTag cells. GLUTag cells were incubated for 2 h in the presence of T-3601386 (0.1–10 μmol/L) or AM-1638 (1 or 10 μmol/L), and secreted active GLP-1 levels were measured by ELISA. Veh; vehicle. Each data point represents mean (N = 2) and each value was showed as a white diamond.
(TIF)

S2 Fig Effects of T-3601386 on glucose tolerance in diabetic N-STZ-1.5 rats. Vehicle, T-3601386 (10 mg/kg) or fasiglifam (10 mg/kg) were orally administered 1 hour before glucose loading, and plasma glucose and insulin were monitored in N-STZ-1.5 rats. Graphs showed the time-dependent changes of plasma glucose **(A)** and insulin **(C)** after the drugs administration. Areas under the curve of plasma glucose and insulin (0–120 min) were shown in **(B)** and **(D)**, respectively. Veh; vehicle, T-386; T-3601386, Fasi; fasiglifam. Each data point represents the mean ± S.D. (N = 6). §p<0.05, §§p<0.01 vs. vehicle by Steel's test.
(TIF)

S1 File Data for Fig 1. (DOCX)

S2 File Data for Fig 2. (DOCX)

S3 File Data for Fig 3. (DOCX)

S4 File Data for Fig 4. (DOCX)

S5 File Data for Fig 5. (DOCX)

S6 File Data for Fig 6. (DOCX)

S7 File Data for S1 Fig. (DOCX)

S8 File Data for S2 Fig. (DOCX)
