# Supplementary figures and images for: GPR40 full agonism exerts feeding suppression and weight loss through afferent vagal nerve
Source: PLoS One. 2019 Sep 16;14(9):e0222653. doi: 10.1371/journal.pone.0222653 (PMC6746387; doi:10.1371/journal.pone.0222653)

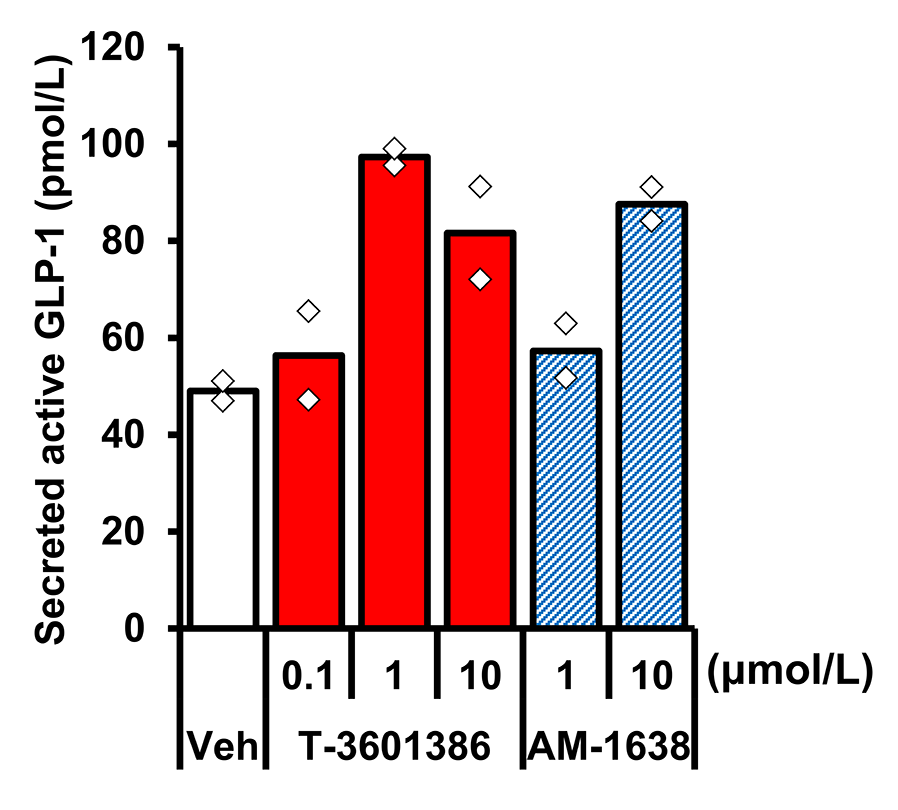

Supplement: S1 Fig — GLUTag cells were incubated for 2 h in the presence of T-3601386 (0.1–10 μmol/L) or AM-1638 (1 or 10 μmol/L), and secreted active GLP-1 levels were measured by ELISA. Veh; vehicle. Each data point represents mean (N = 2) and each value was showed as a white diamond. (TIF) [file pone.0222653.s001.tif]

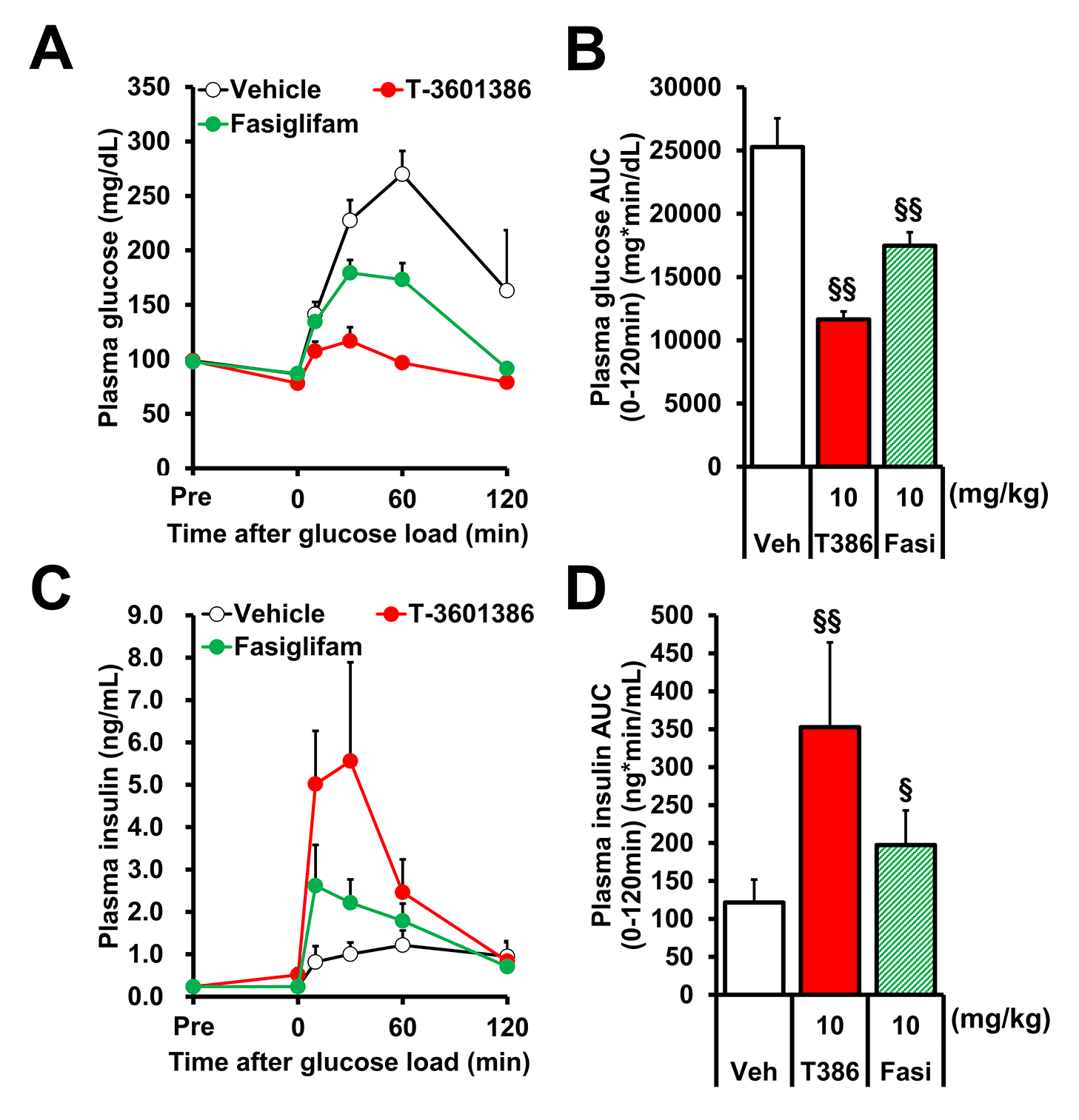

Supplement: S2 Fig — Vehicle, T-3601386 (10 mg/kg) or fasiglifam (10 mg/kg) were orally administered 1 hour before glucose loading, and plasma glucose and insulin were monitored in N-STZ-1.5 rats. Graphs showed the time-dependent changes of plasma glucose (A) and insulin (C) after the drugs administration. Areas under the curve of plasma glucose and insulin (0–120 min) were shown in (B) and (D), respectively. Veh; vehicle, T-386; T-3601386, Fasi; fasiglifam. Each data point represents the mean ± S.D. (N = 6). §p<0.05, §§p<0.01 vs. vehicle by Steel's test. (TIF) [file pone.0222653.s002.tif]
