# Supplementary material for: GPR40 full agonism exerts feeding suppression and weight loss through afferent vagal nerve
Source: PLoS One. 2019 Sep 16;14(9):e0222653. doi: 10.1371/journal.pone.0222653 (PMC6746387; doi:10.1371/journal.pone.0222653)
Supplement: S3 File — (DOCX) [file pone.0222653.s005.docx]

Fig 3A, B

Fig 3C

Fig 3D, E
